# Draft Genome Sequence of Blood-Origin Pasteurella canis Strain PA42, Isolated from a Dog in Japan

**DOI:** 10.1128/mra.00260-22

**Published:** 2022-05-31

**Authors:** Takahiro Maeda, Haruno Yoshida, Jung-Min Kim, Yuzo Tsuyuki, Goro Kurita, Jae-Seok Kim, Takashi Takahashi

**Affiliations:** a Laboratory of Infectious Diseases, Graduate School of Infection Control Sciences, Ōmura Satoshi Memorial Institute, Kitasato University, Tokyo, Japan; b Department of Laboratory Medicine, Kangdong Sacred Heart Hospital, Hallym University College of Medicine, Seoul, Republic of Korea; c Division of Clinical Laboratory, Sanritsu Zelkova Veterinary Laboratory, Tokyo, Japan; University of Maryland School of Medicine

## Abstract

We report the draft genome sequence of Pasteurella canis strain PA42, which was isolated from the blood of a diseased dog in Japan in 2021. The 2.151-Mbp genome has a G+C content of 36.6%. Sequences unmapped to the reference genome sequence of NCTC 11621^T^ (GenBank accession number UGTV00000000.1) were characterized.

## ANNOUNCEMENT

**FIG 1:**
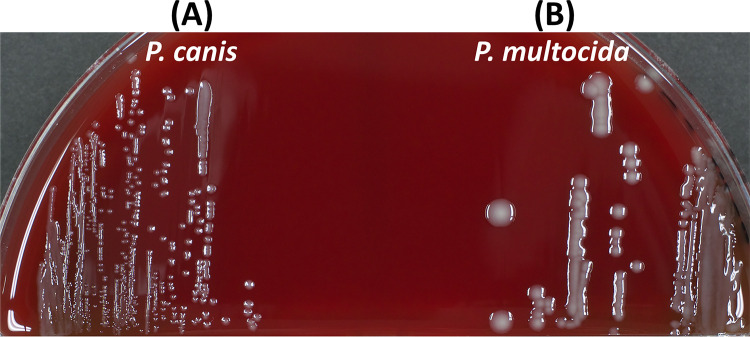
P. canis strain PA42 forms small, smooth colonies (A), compared to P. multocida strain PA17 colonies (B), on a blood agar plate. The strains were inoculated onto blood agar plates and incubated in 5% CO_2_ at 37°C for 24 h.

Pasteurella canis (Pasteurella multocida biotype 6), which was reclassified in 1985 based on DNA homology (1), is a tiny Gram-negative coccobacillus. It grows on blood agar plates as small, smooth colonies, compared to P. multocida colonies (Fig. 1). We report a P. canis genome sequence.

Sanritsu Zelkova Veterinary Laboratory Ethics Committee approved our study design (approval number SZ20220420). Using the Versa TREK blood culture system (Kohjin Bio, Japan) (2, 3), strain PA42 (*Pasteurella*/registered number) was isolated from the blood of a diseased male Saint Bernard in Japan (Okayama Prefecture) in 2021. This isolate was inoculated onto blood agar and incubated in 5% CO_2_ at 37°C for 24 h (Fig. 1). A single colony was grown overnight in Todd-Hewitt broth with yeast extract. The bacterial pellets recovered from 2 mL broth by centrifugation at 3,790 × *g* at 20°C for 5 min were washed twice with phosphate-buffered saline. The final pellet was resuspended in ATL buffer (Qiagen, Germany); RNase A (2 μL, 10 μg/mL) was added to the suspension and incubated at 37°C for 1 h, followed by incubation with proteinase K (20 μL) at 56°C for 45 min. AL buffer (200 μL) was then added and gently mixed. Ethanol-precipitated DNA was resuspended in 0.1× Tris (1 mM)-EDTA-2Na (0.1 mM) buffer. The DNA sample was stored at −70°C to −80°C until it was used. A sequencing library was generated using the TruSeq DNA PCR-free library kit (Illumina, USA). Sequencing was performed using an Illumina NovaSeq 6000 platform with 150-bp paired-end reads. Sequence adaptors and low-quality reads were filtered using Trimmomatic v.0.39 (4).

Sequencing yielded 42,633,052 reads (6,437,590,852 bases). *De novo* assembly was performed using Unicycler v.0.4.8 in default mode, quality scores were assigned to each bridge, and the most supportive bridge was selected. Multiple rounds of Unicycler polishing improved the sequence accuracy. Genome sequences were annotated using DDBJ Fast Annotation and Submission Tool (DFAST) (https://dfast.nig.ac.jp) (5). Assembly metrics and annotated features included the following: genome size, 2,151,012 bp; number of contigs, 28; genome coverage, 2,992×; *N*_50_ value, 177,619 bp; number of coding DNA sequences (CDSs), 1,968; number of tRNAs, 49; number of rRNAs, 3; number of clustered regularly interspaced short palindromic repeats (CRISPRs), 3; G+C content, 36.6%; coding proportion, 89.4%.

Mapping of PA42 reads to the P. canis NCTC 11621^T^ (GenBank accession number UGTV00000000.1) reference genome sequence was performed using the reference tool with default parameters in CLC Genomics Workbench v.22.0. *De novo* assembly using the remaining unmapped reads (1,299,534 reads) yielded 41 contigs. These contigs were uploaded into the Web-based applications PathogenFinder v.1.1 (https://cge.cbs.dtu.dk/services/PathogenFinder) (6) and DFAST to identify any pathogenic gene families that were not present in NCTC 11621^T^. One CDS (903 bp) that was recognized by both applications encodes a phage-related protein (contig 23 [nucleotide start position, 6635; nucleotide end position, 5733]) similar to that of Staphylococcus aureus subsp. *aureus* (GenBank accession number CP002120.1). This sequence in the phage-related region (24.5k bp) of contig 23 was also detected by the phage detection application PHASTER (https://phaster.ca) (7, 8).

### Data availability.

The P. canis genome sequence has been deposited in DDBJ/EMBL/GenBank under the accession number BPUX00000000.1, with the SRA accession number DRR352430.
